# Supplementary material for: Environmental and Genetic Determinants of Serum 25(OH)-Vitamin D Levels during Pregnancy and Early Childhood
Source: Children (Basel). 2019 Oct 21;6(10):116. doi: 10.3390/children6100116 (PMC6826446; doi:10.3390/children6100116)
Supplement: Supplementary file 1 [file children-06-00116-s001.pdf]

Methods

Blood Sample Analysis

After collection, the blood samples were centrifuged for 10 min at 4300 rpm to separate serum, subsequently frozen at −80 °C until analysis. The serum samples were transported on dry ice for duplicate analyses for 25(OH)-vitamin D2 and 25(OH)-vitamin D3 at the Dept. of Clinical Biochemistry, Aarhus University Hospital, Denmark. Serum 25-hydroxyvitamin D levels were analyzed by isotope dilution liquid chromatography-tandem mass spectrometry (LC-MS/MS) [1,2]. Calibrators traceable to NIST SRM 972 (Chromsystems, DE) were used. Mean coefficients of variation (CV) for 25(OH)-vitamin D3 were 6.4% and 9.1% at levels of 66.5 and 21.1 nmol/L and for 25(OH)D2 the CV values were 8.8% and 9.4% at levels of 41.2 and 25.3 nmol/L. The average of the combined 25(OH)-vitamin D values was calculated and used in the analysis. If both 25(OH)-vitamin D2 and 25(OH)-vitamin D3 were under the detection level, the combined value was defined as equal to 10 nmol/L.

Table S1. Baseline characteristics of pregnant women in $COPSAC_{2010}$.

Variables	Week 24 Gestation *n* = 738	1 Week Postpartum *n* = 284
Caucasian, N (%)	665 (95.7)	271 (95.4)
Maternal BMI in kg/m², mean (SD)	24.5 (4.34)	24.6 (4.34)
Gestational age in weeks, mean (SD)	39.5 (1.69)	39.4 (1.59)
Caesarian section, N (%)	150 (21.6)	59 (20.8)
Mother's age at birth in years, mean (SD)	32.2 (4.50)	32.0 (4.28)
SNP rs4588, mean (SD)	1.44 (0.62)	1.44 (0.60)
SNP rs7041, mean (SD)	1.13 (0.70)	1.16 (0.71)
Season of blood sample, N (%)		
Spring	146 (19.8)	63 (22.2)
Summer	164 (22.2)	57 (20.1)
Autumn	233 (31.6)	70 (24.6)
Winter	195 (26.4)	94 (33.1)
Maternal asthma, N (%)	184 (26.6)	76 (26.9)
Maternal smoking in third trimester, N (%)	25 (3.6)	14 (4.9)
Social circumstances PCA score, mean (SD)	0.00 (1.00)	−0.07 (0.94)
Dietary vitamin D intake in µg/d, mean (SD)	4.81 (2.81)	4.71 (2.61)

Table S2. Baseline characteristics of the children in $COPSAC_{2000}$.

Variables	Cord Blood *n* = 257	4 Years *n* = 298
Male, N (%)	124 (48.2)	146 (49.0)
Caucasian, N (%)	249 (96.9)	288 (96.6)
BMI in kg/m², mean (SD)	12.85 (1.30)	15.68 (1.15)
Gestational age in weeks, mean (SD)	40.02 (1.53)	39.91 (1.62)
Caesarian section, N (%)	50 (19.5)	-
Maternal age at birth in years, mean (SD)	29.84 (4.26)	30.34 (4.32)
Genetic score	4.78 (1.75)	4.76 (1.70)
Season of blood sampling, N (%)		
Spring	56 (21.2)	60 (20.1)
Summer	74 (28.8)	79 (26.5)
Autumn	67 (26.1)	93 (31.2)
Winter	60 (23.3)	66 (22.1)
Older siblings at birth, N (%)	106 (41.4)	119 (40.3)
Maternal smoking in third trimester, N (%)	41 (15.95)	-
Nicotine hair level in ng/mg, mean (SD)	-	188.14 (108.48)
Asthma status in third trimester		
Better	79 (30.9)	83 (27.9)
Unchanged	123 (48.0)	155 (52.0)
Worse	54 (21.1)	60 (20.1)
Social circumstances PCA score, mean (SD)	0.00 (0.97)	0.04 (0.95)

References

1. Højskov, C.S.; Heickendorff, L.; Møller, H.J. High-throughput liquid-liquid extraction and LCMSMS assay for determination of circulating 25 (OH) vitamin D3 and D2 in the routine clinical laboratory. *Clin. Chim. Acta* **2010**, *411*, 114–116.
2. Maunsell, Z.; Wright, D.J.; Rainbow, S.J. Routine Isotope-Dilution Liquid Chromatography-Tandem Mass Spectrometry Assay for Simultaneous Measurement of the 25-Hydroxy Metabolites of Vitamins D2 and D3. *Clin. Chem.* **2005**, *51*, 1683–1690.
